# Pathological Anatomy

**Published:** 1861-05

**Authors:** John H. Packard

**Affiliations:** Philadelphia


### PATHOLOGICAL ANATOMY.

BY JOHN H. PACKARD, M.D., OF PHILADELPHIA.

### I.—*Encephaloid Tumors on both sides of the Neck, with Obliteration of the Left Internal Jugular Vein.* By Mr. Cutler.

John T., aged thirty-six, was admitted into St. George's Hospital in October, 1860, with "a large swelling on both sides of the neck, under the jaw, which proved to be medullary cancer. He died from the effects of this on the fifth of November.

"*Autopsy, thirty-one hours after death.*—On dissection, the swelling of the neck was found to depend on a series of globular tumors, apparently generated in the cervical glands, of the soft malignant (encephaloid) variety, filling all the upper part of the neck, resting on the cervical vertebræ behind on both sides, and nearly meeting in front across the middle line. The skin was not adherent. On the right side, the carotid artery, pneumogastric nerve, and internal jugular vein passed completely through the tumor. The vessels were widely separated, but were quite pervious. The nerve did not present any obvious appearance of injury. On the other side, the vessels also passed through the tumor, and were less widely separated; but the vein was completely impervious at its upper part. The tumor presented a whitish uniform section, and contained an abundant creamy juice, in which multitudes of large nucleated cells of various forms, many of them containing oil-globules, were seen. On the left side, large masses lay over the angle of the jaw, and others were found passing behind the hamular process, and pressing on the floor of the mouth. The parts about the left tonsil seemed sloughy, though the tonsil itself was natural. The pharynx and larynx were healthy. The sterno-mastoid muscle on each side was lost in the tumor. The veins at the root of the neck were distended with blood. The right side of the heart was loaded with decolorized blood. There were old pleuritic adhesions. The heart, lungs, and abdominal viscera were healthy."—(*Lancet*, December 29, 1860.)

II.—*Rupture of the Lung without Fracture of the Ribs.* By M. MARJOLIN.

A boy, aged thirteen and a half years, was brought to the Hôpital Sainte-Eugénie, December 3d, 1860, having had his chest run over by the wheel of a dray. His dyspnœa was excessive, but there was no hæmoptysis, hæmatemesis, subcutaneous emphysema, or pain in the belly; there was a contusion on the front of the chest, but no fracture of the ribs could be detected. On auscultation, the natural sounds were heard all over the left side of the thorax, but on the right side there was amphoric breathing, with metallic tinkling; the dyspnœa prevented percussion over this side. Death occurred on the third day. "On post-mortem examination, the right lung was found to be ruptured in two places. One rupture was situated near the anterior margin of the upper lobe, and was nearly half an inch in depth, the other was at the junction of the upper with the middle lobe. The pleural cavity contained eight or nine ounces of blood, and the surfaces of the lung and of the opposed wall of the chest were covered by layers of recent lymph of considerable thickness. The right lung and pleura were perfectly normal. The third and fourth left ribs, and the third right rib were incompletely fractured. It was only the external surface which was fractured, and the corresponding pleural surface was quite intact."—(*Gazette des Hôpitaux*, Dec. 29th, 1860.)

A very similar case is recorded by Mr. Bryant, in the *Guy's Hospital Reports*, (3d series, vol. vi. p. 43.)—(*British Medical Journal*, Feb. 2, 1861.)

III.—*Patency of Foramen Ovale.* Reported by DR. BOWDITCH to the Boston Society for Medical Improvement.

This lesion occurred in the person of a lady, who until she was nineteen years of age had been unusually strong and agile. At that time she noticed some dyspnœa from dancing, while at a ball. She married a few years afterward; but never bore any children. Without any symptoms of disease about the heart, or serious lesion of the lungs, her digestive, renal, and uterine functions being likewise perfect, she suffered more and more from difficulty and oppression in breathing, and died at the age of 45.

"At the *autopsy*, the right cavities of the heart were found much hypertrophied; the left were normal, or nearly so. The foramen ovale, an inch in diameter, was round and smooth, with a thin edge. All the valves were perfectly normal. The lungs had old tuberculous disease to a small extent, in both apices. Owing to circumstances beyond control, the other organs were not specially examined, but they seemed normal."—(*Boston Medical and Surgical Journal*, Feb. 28, 1861.)

IV.—*Aneurism at the Apex of the Left Ventricle of the Heart.* Reported by Mr. Canton to the Pathological Society of London.

A gentleman, aged forty-nine, died suddenly during the night. He was of large and muscular frame, and had always enjoyed good health.

Masses of fat were found nearly concealing the pericardium from view. Intimate adhesions existed between the opposed surfaces of the membrane over nearly its whole extent. The left ventricle was thickened except toward the apex, where a dilatation large enough to contain a plover's egg had occurred. Pericardium, degenerated muscular tissue, and endocardium formed the walls of the sac; the latter of these layers was thickened and opaque, and had firmly attached to it some flakes of old and laminated fibrin. The thickening and opacity extended above the neck of the sac, which was wide.—(*Lancet*, January 12, 1861.)

V.—*Aneurism of the Aorta, causing by its Pressure such Destruction of the Spinal Vertebræ as nearly or quite to lay open the Medullary Canal.* Reported by Dr. John Ogle to the Pathological Society of London.

The subject of this disease was a patient under the care of Dr. Seymour, who has related the case in his work on the *Nature and Treatment of Dropsy.*

When dried and prepared, the aneurismal sac measured five and a half inches by four and a half, commencing about an inch below the left subclavian, and extending nearly to the diaphragm. The last seven dorsal and the first lumbar vertebræ had suffered from its pressure, caries of their transverse processes and pedicles, and of the adjacent parts of several ribs, having taken place. Some distortion of the column had occurred at this point. "*Three of the ribs were found completely broken up into several portions, which were lying loose in the coagula of the sac.*" In front, the sac was indented by the pressure of the œsophagus. At three points the sac communicated with that of the medullary canal, the openings having probably been plugged, when recent, with firm clots of blood. One of these openings was about an inch and a half long by half an inch broad; the two others were nearly circular, and about the size of a ten-cent piece.—(*Transactions of the London Pathological Society*, vol. xi.)

VI.—*Aneurism of the Ascending Aorta.* By Dr. Ogle.

A woman, aged forty, was admitted into St. George's Hospital, having noticed, ten months before, a swelling between the third and fourth ribs, a little to the right of the sternum; the symptoms showed this to be an aneurism. Three weeks later, crowing inspiration, orthopnœa, and frothy, muco-purulent expectoration were observed; both pupils were somewhat dilated. In a few days the right pupil became very much dilated; the right arm became œdematous, the breathing very labored, and the pulse very feeble, until death took place.

A *post-mortem* examination showed that the sac "had given way, permitting its contents to come in contact with the pectoral muscles. Portions of these muscles and of the sternum were much absorbed, and *the sternal end of the third rib was lying loose in the aneurismal cavity.* The sac, which was of the size of a cocoanut, communicated by a circular, smooth-edged aperture, of the size of half a crown, with the upper part of the ascending aorta; and the aorta itself, from its commencement to where it became thoracic, was dilated to the size of a closed, moderately-sized fist, having much atheromatous substance in its walls." The other organs were healthy.—(*Ibid.*)

[In the American Journal of the Medical Sciences for July, 1858, I reported a case in which portions of the first piece of the sternum were found by me in the cavity of a very large aneurism of the aortic arch. Such occurrences are extremely rare.—P.]

VII.—*Aneurism of the Lower Part of the Thoracic Aorta, bursting into the Substance of the Right Lung.* By DR. OGLE.

This patient, a man aged fifty-four, had the usual symptoms of aneurism; he referred his illness to a strain of his back from lifting, a year before he came under notice. He was treated in St. George's Hospital for some time, and then went away; he eventually died of pneumonia, accompanied by hæmoptysis.

At the autopsy, "an aneurism, about twice the size of a cricket-ball, was found at the lower part of the thoracic aorta. This communicated with a bilocular cavity in the base of the right lung, the general substance of which was greatly disintegrated. The cavity in the lung communicated with one of the minute bronchial tubes. The coats of the aorta, generally, were the seat of calcareous and atheromatous deposit, and the arch of the aorta was dilated. The heart itself was large and fatty, its walls being thinned, and the left auriculo-ventricular orifice was dilated."

Dr. Ogle remarks upon the rarity of the termination of aneurism, and upon the evidence in favor of the possibility of the disease being due to muscular straining.—(*Ibid.*)

VIII.—*Aneurism of the Aorta.* By PETER EADE, M.D., etc., Physician to the Norfolk and Norwich Hospital.

A stout, fat man, aged fifty-two, died in the hospital, after suffering from more or less distinct symptoms of thoracic aneurism for about seven months. He observed blood in his expectoration and stools some weeks before his death, which was immediately preceded by the coughing up of a large quantity of nearly pure scarlet blood.

"*Body examined forty hours after death.*—On raising the sternum, a tumor was seen beneath its upper part, which adhered to this bone and to the left upper ribs. The left external jugular vein was involved in it, and its cavity obliterated. The trachea was seen to be pushed over to the right of the median line. From one to two pints of clear yellowish serum were found in the left pleural sac. The right lung was enormously distended; its lobules very plainly marked out on its surface, crepitant everywhere, and when cut into, quickly collapsing. The left lung was collapsed, solid, and infiltrated throughout with dirty white or grayish purulent matter, and with lymph not yet converted into pus. Blood was found in the mouth and pharynx, but not in the trachea. On opening this tube, the cartilages of the larynx were found to be rigid and ossified. The left bronchus was flattened and compressed, but not quite closed, and about half an inch above its orifice in the wall of the trachea was seen an irregularly-rounded, ulcerated opening, the size of the end of the little finger, which opening was found to communicate with the cavity of an aneurismal sac, through a mass of coagulated fibrin contained in its interior. The œsophagus in its upper part was empty. Just below its middle it was encroached upon by a bulging swelling of the size of a small orange, and just below this again was contracted to a very small size, but healthy. Coagulated blood was found in it both above and below the swelling. The heart was covered with much fat externally. Its walls were thin, and all its cavities enlarged. The muscular tissue of both right and left ventricles was found (under the microscope) to be in a state of advanced fatty degeneration; the valves were healthy. The aorta was greatly diseased; its lining membrane closely studded with atheromatous and bony scales and plates. These patches covered the greater part of its inner surface near the heart, and were very numerous and distinct even into its abdominal portion; but the large arteries of the neck, up to their junction with the aorta, were entirely free from the disease. Two aneurismal sacs were found to spring from the aortal arch. One, the smaller, about the size of a duck's egg, sprang from its upper part, between (but not involving) the great arteries. It appeared to be composed of all the arterial tunics, was half filled with fibrinous coagulum, and communicated with the artery by an aperture so large as virtually to make it a portion of this tube. The other, the larger, also a true aneurism, and also opening into the aorta by an orifice nearly as large as the entire diameter of the sac, arose from the back part of the arch, immediately behind the left subclavian artery. It formed the large bulging seen in the œsophagus, and through the layers of fibrin contained in its interior blood had escaped by the ulcerated opening into the trachea. The left pneumogastric nerve was seen crossing over the front of the aorta to enter the left lung. It was much flattened, (and must have been considerably stretched,) and one large branch, just prior to its entrance into the lung, so much so as to exhibit quite an acute edge."

This case is remarkable in several ways. In the first place, the existence of two distinct aneurismal sacs, taking different directions, pressing upon different organs, and neither of them giving rise to physical signs. Secondly, the occurrence of hemorrhage three weeks before the fatal issue of the disease. Thirdly, the destructive inflammation of the left lung, probably in consequence of the impeded functions of the corresponding pneumogastric nerve. Fourthly, the fact that this patient was decidedly benefited by a stimulating plan of treatment, while his sufferings were aggravated by depletion.—(*Lancet*, February 16, 1861.)

IX.—*Rare Case of Diseased Liver.* By S. Martyn, M.D., etc.

J. P., aged thirty-eight, was admitted into the Bristol General Hospital, November 7th, 1859, suffering from ascitic distention of the abdomen, for which he had been tapped. His habits had been very irregular. Tapping was again performed on the seventeenth of November, and a third time on the fifth of January, 1860. On the day after the last operation he died.

"*Post-mortem examination.*—The organs were generally healthy, except the liver and peritoneum; the latter showing traces of inflammation of slight and recent character. The liver was somewhat large, its surface being marked by a few depressed lines, and covered with recent lymph. On section, its lobules appeared either healthy and their cells not overloaded with fatty granules, or else obscured by purple congestion. The gall-bladder was empty. At the centre of the liver there was the following state of things near the transverse fissure, which was occupied around its vessels, etc., by a hard fibrous mass. On section here the knife encountered scirrhous portions, evidently composed of liver tissue altered by a new and fibrous ingredient. Hence the pale-red substance was close in texture, very elastic, and hard; while vessels passing through it were obliterated in the transit, and pervious further on. In the centre of the hardest parts the knife passed through white or very faintly yellowish masses of softer matter, the sections of which, to the number of three or four, varied up to an inch and a half in diameter. These whitish masses were surrounded by delicate zones of redness, and situated in these incomplete cysts. Irregular in shape, they might be turned out of their hollows by tearing some adhesions; when it appeared that they were in the tracks of portal canals, though, having burst them up, they excavated the liver tissue itself. Through one of these white masses an occluded portal vein ran; but some large portal trunks remained patent. The interiors of the emptied hollows were mottled with yellow matter. A probe would not pass up the cystic duct from the empty gall-bladder. The material of the yellowish-white masses was quite homogeneous, very elastic, and tore like white of egg boiled very hard. A microscopic section, from wherever taken, showed the following elements: 1. A granular base not influenced by ether. 2. Refracting granules soluble in ether. 3. Wrinkled spheroidal cells, averaging $\frac{1}{3000}$ of an inch in diameter, and possessing, when acted on by acetic acid, very much the character of pus cells. 4. Small plates of cholesterine. 5. Minute granular masses of orange-colored biliary matter. 6. Masses of radiating acicular crystals. 7. Some columnar epithelium. Alcohol, in which a piece had been boiled, soon deposited cholesterine, and, when evaporated to dryness, concentric scales resembling leucine. Nitric acid applied to a slice indicated protein and bile-compounds, producing also some effervescence. The star-shaped bodies were composed of tyrosin, as far as I could determine, the quantity present sufficing for microscopic analysis only. Insoluble in ether, and almost so in alcohol, they were readily taken up by mineral acids, and destroyed by heat. On boiling pieces of the white substance in water, filtering and concentrating, there were deposited beautiful stars of crystalline needles, possessing most of the characters of tyrosin. The pale-red condensed liver tissue around the white masses proved a fine example of abnormal development of connective tissue. It was crowded with large oval nuclei; and, at the torn edge of the preparation, each was seen to belong to a fusiform fibre-cell with dividing extremities.

"The cause of death in this person was evidently an obstruction to the portal circulation, occasioned by the diseased tissue in and around the transverse fissure of the liver. The morbid appearances were due doubtless to a previous state of inflammation; for the thickened areolar tissue in the transverse fissure, the condensed tissue of the liver itself in the neighborhood, and the hard cicatrix-like metamorphosis of parts near the white masses, all point to the inflammatory condition. The white, imperfectly encysted masses must have arisen out of this process; for the cells in them could have come only from the epithelium lining the biliary ducts, and thrown off, after the manner of pus-cells, from any irritated mucous surface. The white masses are pervaded by elements of bile, which must therefore have been freely mixed with the cells in the dilating ducts while the whole was semifluid. That certain ducts should have been dilated in this way is to be attributed to their occlusion by fibrous constricting tissue. Other ducts, remaining unaffected, conveyed the bile from the rest of the liver, so that its function, *quoad* the bile, was not appreciably impaired.

"The pathological sequence would probably be as follows: 1. Irritating matter, present in the portal trunks. 2. Irritation set up in their neighborhood, ending in rapid cell-growth of the connective tissue, and its condensation. 3. Irritation of the ducts involved, and cell-growth in them, with admixture of bile (that mere occlusion of the ducts will not produce this effect is proved by cases recorded by Bright and Frerichs.) 4. Solidification of this semifluid mass, now cut off below by a fibrous structure. 5. Gradual constriction of portal canals and veins by cicatrix-like contraction, occasioning venous congestion, dropsy, deprivation of nutritive matter, and death."

Deposits of this kind have been described as cancerous or tubercular; they have been called "tubera," "retrograde cancer," "encysted knotty tumors," and obsolete abscesses. Dittrich first traced the connection between them and the syphilitic poison, which may perhaps be directed toward the liver in consequence of the habit of spirit drinking.—(*British Medical Journal*, Feb. 2, 1861.

X.—*Marked Icterus, with Fatty Degeneration of the Liver and Kidneys.* By Dr. Von Plazer.

Marie St. ——, aged twenty-six, an unmarried servant girl, of good constitution, came under treatment in October, 1859. She had suffered for two or three months with constipation and digestive disorder, accompanied with colicky pains in the abdomen, particularly in the epigastrium: they came on in periodical accessions. Two days before she applied for medical advice she had observed that she was jaundiced, and began to be troubled with malaise, headache, heat, and vertigo, obliging her to give up work.

Her skin and conjunctivæ were moderately tinged yellow, her tongue foul, and her abdomen slightly meteoric; the spleen was swollen, the skin hot and dry, pulse and respiration a little quick, the urine scanty, brownish yellow, cloudy, and albuminous; stools, pale brown and pasty. The chief subjective symptoms present were somnolence, dullness, vertigo, sensitiveness of the liver and stomach upon pressure, nausea, thirst, and loss of appetite.

All these symptoms were aggravated on the next day, when she passed into a state of alternating coma and delirium. On the third day of the treatment she died.

*Autopsy.*—The skin was still tinged yellow, and the abdomen meteoric. The cerebral meninges were œdematous, the brain swollen, congested, infiltrated with serum; at its base were about two ounces of bloody serum. The lungs were somewhat œdematous; the heart was flaccid, and numerous ecchymoses existed beneath the endocardium and pericardium.

The liver was swollen and heavy, its left lobe and the left half of its right lobe colored deep yellow, the remainder of it pale brown; its substance was soft and friable, anæmic, greasy to the knife; the lobuli were indistinct. The gall-bladder was small, containing a little clear yellow bile; the ducts pervious.

The spleen was enlarged one-half, its capsule tense, its parenchyma soft and deep red. The stomach and intestines were a good deal distended with gas, the mucous membrane puffy, dotted with ecchymoses of the size of a pin's head, and covered with deep-brown thick mucus. The kidneys were large, flaccid, somewhat yellow, moderately injected; the cortical part pale yellow, here and there streaked with red, the medullary part brownish red. The urinary bladder was small, and contained three or four ounces of cloudy, icteric urine.

On microscopical examination, the liver was found in a state of extreme fatty degeneration. The kidneys were in a less advanced stage of a similar condition.

M. Von Plazer is disposed to regard this case as one of acute atrophy, the fatty change becoming rapidly developed. He quotes similar cases published by Rokitansky in the *Zeitschrift der Wiener Aerzte* for 1860, but objects to an idea suggested by this observer, that uræmic poisoning may be the proximate cause of death.—(*Gazette Hebdomadaire*, Dec. 21, 1860; from *Prager Vierteljahrschrift* for 1860, t. ii.)

XI.—*Cancer of the Gall-bladder and neighboring Tissues.* By T. W. BELCHER, M.A., etc.

Dennis Donovan, aged about fifty, had been a hard drinker in earlier life. He was for some time an inmate of the hospital of the Cork workhouse, suffering chiefly from constipation of the bowels and sickness of stomach; he was much emaciated, but his belly was tumid, and in some places nodulated. Death seems to have occurred from exhaustion; an autopsy was made twenty-four hours afterward.

"So thin were the abdominal parietes, that the first touch of the knife penetrated the colon, when a large quantity of feculent matter, of the most offensive odor, shot up with much force, and continued to exude so long as the examination lasted. On opening the thorax, a most horrible discharge of fetid gas took place, so that all concerned had to leave the room instantly.

"The lungs were dark and congested; the heart natural, and the thoracic viscera, in other respects, healthy. The liver was of the natural size, and apparently healthy. The gall-bladder was replaced by a cancerous tumor, the entire contents of the lesser omentum being one cancerous mass. The posterior surface of the mass was nodulated, and contained gall-stones in numbers. The ascending colon was large—so large as to resemble the stomach in general appearance; the junction of the ascending with the transverse portion was involved in the disease, and so strictured that the finger could be passed through it with difficulty. The right kidney was enlarged, its pelvis cancerous, and the whole involved in the diseased mass." The left kidney was normal.—(*Dublin Quarterly Journal of Medical Science*, Feb., 1861.)

XII.—*On the Development of the Uterine Glands in Sarcomata (Fibrous Tumors) of the Uterus and Ovaries.* By PROFESSOR ROKITANSKY.

These tumors are closely allied, in anatomical composition, with certain alterations occurring in the mucous membrane and submucous tissue of the womb in the course of chronic uterine catarrh. In the latter affection the mucous membrane sometimes presents a spongy areolar or granulated swelling, due to hypertrophy of the tissue lying between the uterine glands, which are themselves elongated or distended. Sometimes the tissue and the glands are equally hypertrophied; and this hypertrophy, if circumscribed, may at length give rise to a veritable pedunculated polypus, in which the glands are at last wholly or in part obliterated. In the latter case, their remains sometimes become filled with gelatiniform mucosity or colloid matter, are dilated, and there is finally constituted a mass of small rounded or polyhedral cysts, imbedded in a stroma of connective tissue; these are the so-called *cellular* polypi, in which the superficial cysts sometimes burst, empty themselves, and are replaced by normal cysts.

In some cases, the elongation of the glands occurs not only in the direction of the uterine cavity, but also outward, from the submucous tissue to the tissue proper of the uterus, in the substance of which they form thus a species of plug of fibrous aspect.

Sometimes, again, the hypertrophy of the submucous tissue ends in the development of fibrous tumors, within which are found not only vestiges of elongated glands, but also pouches of new formation, which at last give rise to the development of small cysts.

As derivatives from this form, we find certain fibrous polypi of the uterus which contain glandular pouches; these may arise from the normal glands, but they are sometimes of new formation. They are found, indeed, in the substance of large polypi, at a great distance from the mucous surface. These pouches may at last undergo the changes before mentioned, and especially they may become cysts. It may then happen that the tissue of the fibrous mass sprouts out into the interior of the cysts in the form of papillary excrescences. Upon section, such a body would present a cracked and granular aspect. What takes place here is an exact repetition of what we may observe in the development of adenoid tumors of the breast. Hence these are in fact *adenoid tumors* of the uterus. Such tumors are met with also in the ovary, with all the characters proper to the uterine glands; which places beyond a doubt the possibility of a formation *de novo* of such glands.—(*Gazette Hebdomadaire,* from *Zeitschrift der Gesellschaft der Aerzte zu Wien,* 1860, No. 37.)

XIII.—*Singular Case of Hermaphrodism.* By Henry J. Buck, Esq., M.R.C.S.

The subject of this notice had for seventy-three years passed for a female, but was of powerful frame, and manifested sexual passions toward women. She once, in London, assumed men's clothes, and even engaged in boxing-matches. She was five feet ten in height, had some beard, no development of mammæ, and a gruff, bass voice. Her habits had been irregular.

"In the place of the umbilicus an ulcerated surface existed, at the lower part of which two small apertures showed the terminations of the ureters, through which the urine constantly escaped, rendering necessary the continual application of cloths to the part.

"A depression might be observed between the ossa pubis, and, on examination, it was found that there was an arrest of development of these bones, they being nearly three inches apart.

"A mass or outgrowth of skin, composed of three portions—the third being merely a small nodule—occupied the place of the penis. The testicles were found on either side of this apology for a penis, forming a projection resembling in appearance an enlarged inguinal gland. The testes were small, and apparently perfect, and were connected by the before-mentioned mass of skin; but the vasa deferentia could not be accurately traced. The bladder was absent; the anus perfect. The 'penis' was composed of nothing but cellular and connective tissue, the larger portion showing glandular structure, but in no organized form." —(*Proceedings of the Royal Medical and Chirurgical Society*, in the *Lancet*, December 8, 1860.)

XIV.—*Sero-sanguineous Cyst of the Nates.* By M. COULON.

A female child thirteen days old was brought to the Hôpital des Enfants Assistés, having a congenital tumor as large as itself upon its buttocks. The tumor was ovoid, and somewhat flattened antero-posteriorly; it was attached to the child by a thick pedicle, but the line of division was quite evident. At its upper part, in front, the anus was perceptible, opening directly forward; a fold ran backward from it so as to divide the growth into two equal portions. A less distinct depression existed posteriorly. To the touch the sacrum seemed abnormally wide, but no gap could be detected. From the lowest part of the sacrum ran several diverging fibrous cords, easily perceived through the skin; the middle one adhered to the skin, and caused a slight ridge corresponding to its course.

Each half of this bilobed ovoid tumor was nodulated; the skin over some of the nodules being normal, and that over others of a violaceous tint. Two of these latter, elliptical in shape, were situated one on either side of the median line anteriorly; the skin covering them seemed about to give way. Fluctuation was evident throughout the cyst, which did not swell when the infant cried, nor diminish under pressure; it sometimes, however, became less tense than usual. Its circumference in its long axis was over fourteen inches; in its lesser axis, nearly ten. When the child was seated upon it, her feet were over two inches above the supporting plane.

The child died on the nineteenth day. Upon puncturing the cyst, exit was given to about three pints of clear, mahogany-colored, slightly glutinous liquid. A red deposit, which fell to the bottom when this was allowed to stand, was found to consist of blood corpuscles and epithelial cells.

Dissection showed the cyst to be unconnected either with the spinal marrow or with the rectum. It was attached by a pedicle to the coccyx; its thin fibrous wall seemed formed of the distended posterior perineal fascia, and the posterior median cord before alluded to represented the tendon of the sphincter. Its lining membrane was smooth, vascular at some points, and anteriorly pouched like the interior of the wall of the heart. Projecting from its inner surface posteriorly was a small transparent cyst, as large as a nut, full of a clear, limpid serum. The walls were at some points fibrous, at others as thin as paper; microscopically, they consisted of elastic and connective tissue.

This tumor must be regarded as analogous to the spontaneous hæmatoceles which occur elsewhere; as, for instance, in the neck.—(*Gazette Hebdomadaire*, Jan. 18, 1861.)

[Two cases very similar to the one above given were reported to the Pathological Society of Philadelphia by Dr. Keller, in November, 1857. See the Society's *Proceedings*, vol. i. p. 15.—P.]

